# Mobile Health Hearing Aid Acclimatization and Support Program in Low-Income Communities: Feasibility Study

**DOI:** 10.2196/46043

**Published:** 2023-08-23

**Authors:** Caitlin Frisby, Robert H Eikelboom, Faheema Mahomed-Asmail, Hannah Kuper, David R Moore, Tersia de Kock, Vinaya Manchaiah, De Wet Swanepoel

**Affiliations:** 1 Department of Speech-Language Pathology and Audiology University of Pretoria Pretoria South Africa; 2 Virtual Hearing Lab, Collaborative initiative between the University of Colorado and the University of Pretoria Aurora, CO United States; 3 Ear Science Institute Australia Subiaco Australia; 4 Ear Sciences Centre, Medical School, The University of Western Australia Nedlands Australia; 5 Faculty of Health Sciences, Curtin University Bentley Australia; 6 International Centre for Evidence in Disability, London School of Hygiene & Tropical Medicine London United Kingdom; 7 Communication Sciences Research Center, Cincinnati Children’s Hospital Medical Center and University of Cincinnati Cincinnati, OH United States; 8 Manchester Centre for Audiology and Deafness, University of Manchester Manchester United Kingdom; 9 hearX Foundation Pretoria South Africa; 10 Department of Otolaryngology-Head and Neck Surgery, University of Colorado School of Medicine Aurora, CO United States; 11 UCHealth Hearing and Balance, University of Colorado Hospital Aurora, CO United States; 12 Department of Speech and Hearing, School of Allied Health Sciences, Manipal University Manipal India

**Keywords:** community-based rehabilitation, community health care worker, text message, messaging, motivational, reminder, acclimatization, technology use, hearing aid acclimatization, hearing aid, hearing loss, low- and middle-income countries, LMIC, low income, developing country, low resource, hearing, audiology, mobile health, mHealth, health care workers, usability

## Abstract

**Background:**

The most common management option for hearing loss is hearing aids. In addition to devices, patients require information and support, including maintenance and troubleshooting. Mobile health (mHealth) technologies can support hearing aid management, acclimatization, and use. This study developed an mHealth acclimatization and support program for first-time hearing aid users and subsequently implemented and pilot-tested the feasibility of the program. The program was facilitated by community health workers (CHWs) in low-income communities in South Africa.

**Objective:**

This study aimed to evaluate the feasibility of an mHealth acclimatization and support program supported by CHWs in low-income communities.

**Methods:**

An application-based acclimatization and support was adapted and translated for use in low- and middle-income countries. This program was delivered in the form of 20 different voice notes accompanied by graphical illustrations via WhatsApp or 20 different SMS text messages. The program was provided to first-time hearing aid users immediately after a community-based hearing aid fitting in March 2021 in 2 low-income communities in the Western Cape, South Africa. The 20 messages were sent over a period of 45 days. Participants were contacted telephonically on days 8, 20, and 43 of the program and via open-ended paper-based questionnaires translated to isiXhosa 45 days and 6 months after the program started to obtain information on their experiences, perceptions, and accessibility of the program. Their responses were analyzed using inductive thematic analysis.

**Results:**

A total of 19 participants fitted with hearing aids received the mHealth acclimatization and support program. Most participants (15/19, 79%) received the program via WhatsApp, with 21% (4/19) of them receiving it via SMS text message. Participants described the program as helpful, supportive, informative, sufficient, and clear at both follow-ups. A total of 14 participants reported that they were still using their hearing aids at the 6-month follow-up. Three participants indicated that not all their questions about hearing aids were answered, and 5 others had minor hearing aid issues. This included feedback (n=1), battery performance (n=1), physical fit (n=2), and issues with hearing aid accessories (n=1). However, CHWs successfully addressed all these issues. There were no notable differences in responses between the participants who received the program via WhatsApp compared with those who received it through SMS text message. Most participants receiving WhatsApp messages reported that the voice notes were easier to understand, but the graphical illustrations supplemented the voice notes well.

**Conclusions:**

An mHealth acclimatization and support program is feasible and potentially assists hearing aid acclimatization and use for first-time users in low-income communities. Scalable mHealth support options can facilitate increased access and improve outcomes of hearing care.

## Introduction

Hearing loss is one of the most prevalent impairments [1], with estimates showing that more than 2.5 billion persons globally will present with some degree of hearing loss by 2030 [2]. The most common form of rehabilitation to address hearing loss is hearing aids [3]. However, approximately 90% of individuals who could benefit from hearing aids do not use them [4,5]. This situation is even worse in low- and middle-income countries (LMICs), including in the Africa region, where only 2.1% of individuals who could benefit from hearing aids use them [4]. Unaddressed hearing loss has several far-reaching associations, including social isolation [6], depression [7], an increased risk of unemployment [8] or early retirement [9], and increased risk of developing dementia [10]. Furthermore, the global cost of unaddressed hearing loss is estimated at US $980 billion annually [5]. It is therefore important to address the causes of poor hearing aid uptake, such as lack of perceived need, the high costs associated with hearing aids, limited or a total lack of hearing health care services, a lack of awareness of hearing loss, and stigma [11,12].

In recent years, there has been a drive to develop innovative person-centered service-delivery models to make hearing aids more accessible and affordable [5,13-15]. However, the lack of trained professionals to provide these services and support hearing aid users is a significant barrier [5,16-18].

Consequently, the World Health Organization recommends adopting task shifting as a strategy to move ear and hearing services to community health care workers (CHWs) in order to improve the accessibility and availability of hearing services and hearing aids in these contexts [5]. CHWs with minimal training have already provided a range of hearing health care services, including hearing screening [19-21], diagnostic testing [22], and even treatment in the form of hearing aids in both adults and children [23-25]. However, person-centered treatment requires not only the provision of hearing aids but also ongoing information and support, such as assistance with device maintenance and troubleshooting [5]. Most commonly, information regarding hearing aid management is provided to users verbally by an audiologist [26]. However, difficulty retaining information or being provided with insufficient information on hearing aid use may lead to poor management [27-29] and consequently reduced hearing aid satisfaction [30], perceived benefit [31], and use of hearing aids [5,28,32]. Low hearing aid use is an important concern as estimated 20% to 50% of adult hearing aid users either do not make use or only make limited use of their hearing aids [33-35]. Several factors, including hearing aid handling issues, expectations, and counseling, could contribute to the nonuse of hearing aids and should be considered when providing hearing aids to individuals [11,36].

Several studies have used alternative methods to improve the quality and retention of the education and training provided to hearing aid users after fitting to address the factors contributing to nonuse. These methods included home education programs delivered via DVD or videotape recording [37], web-based training and guidance programs via the internet [38,39], telephonic consultations with an audiologist to supplement written information on hearing aids [40], and multimedia educational programs in the form of reusable learning objects via DVDs or the internet [27,41]. Several of these studies could be conducted at the hearing aid users’ homes independently, thus reducing a need for travel to a traditional audiological appointment [37-40]. Most of these studies used videos to enhance the training provided [37-39]. This required users to have access to DVD or video players or a computer, which limits the generalizability to LMICs where these devices are not readily accessible. More recently, a mobile health (mHealth) version of the reusable learning object program, m2Hear, was developed to educate hearing aid users via smartphones [42,43]. mHealth refers to technologies aimed at improving health care services and outcomes using mobile and wireless devices [5]. The use of smartphones increases the accessibility of such a program [42,43]. These studies demonstrated positive outcomes for hearing aid users, indicating that for successful acclimatization they need information on hearing loss, hearing aid orientation, managing expectations, device functionality and maintenance, insertion, device controls, cleaning, communication strategies, adapting to the device, and troubleshooting [27,28,37,38,42]. However, a limitation is that these studies were mainly conducted in high-income countries [44].

Innovative mHealth support programs can provide a potential strategy to facilitate acclimatization, continued or consistent use, and benefit from hearing aids [45]. The combination of task shifting to CHWs supported by mHealth technologies to assist in point-of-care services and remote support is therefore a potentially powerful enabler of community-based care [5,20,46]. This has, however, not been attempted in LMIC settings. This nonexperimental study developed a community-based rehabilitation (CBR) model for providing an mHealth acclimatization and support program for first-time hearing aid users and subsequently implemented and pilot-tested the feasibility of the program. The program was facilitated by CHWs in low-income communities in South Africa. This study aimed to evaluate the feasibility of an mHealth acclimatization and support program supported by CHWs in low-income communities.

## Methods

### Ethics Approval

Institutional review board approval was granted by the Research Ethics Committee, Faculty of Humanities, University of Pretoria (HUM030/0621). Participants were provided with detailed information about the study in their home language, and written informed consent was obtained from all participants before data collection commenced. Participants could keep their hearing aids free of charge once the study ended. Study data were deidentified to ensure privacy and confidentiality.

### Context and Feasibility Framework

This study formed part of a community-based service-delivery model previously reported by Frisby et al [25]. This study, however, focuses specifically on the mHealth acclimatization and support program. The study comprised two phases: (1) development of the mHealth acclimatization and support program designed for LMICs and (2) implementation and pilot testing of this program. This study commenced in March 2021 and took place in 2 low-income communities in the Western Cape, South Africa. It used the Bowen et al [47] feasibility study framework with an implementation science approach for developing and piloting a CBR model for providing an mHealth acclimatization and support program. To determine the feasibility of this program, participant responses to illustrative open-ended questions were collected and analyzed. Positive stakeholder opinions and perspectives and hearing aid use were considered indicators of feasibility.

#### Phase 1—Development of an mHealth Acclimatization and Support Program

##### Developing the Messages

An application-based acclimatization and support program developed by a web-based direct-to-consumer hearing aid provider in the United States (Lexie Hearing) was adapted and translated to isiXhosa for this project through a collaboration with a local nongovernmental organization (NGO; hearX Foundation). The program, originally developed by audiologists for in-app delivery at Lexie Hearing, consisted of 20 unique messages in English, covering various aspects of hearing aid acclimatization and support. The content was adapted for delivery through WhatsApp voice notes or standard SMS text messages.

This program aims to improve the use of hearing aids by providing basic information on how a hearing aid works, optimal use and care, troubleshooting, and motivational support (Table 1) [48,49]. Three authors (CF, TdK, and DWS) reviewed, cross-checked, and discussed disagreements to ensure that appropriate terminology was used in the messages. The Flesch Reading Ease formula was used to ensure that messages were easy to read and understand [50,51]. This readability formula is a web-based tool where messages are imported into a textbox, and the readability and grade-level scores are calculated automatically [52]. The number of words per sentence and the number of syllables per word are used to calculate a readability score that ranges from 0 (cannot be read) to 100 (very easy to read) [50].

**Table 1:** Overview of goals for the mHealth^a^ acclimatization and support program and topics of the 20 messages sent to achieve these goals.

Goal^b^ and message topics			Day message sent
**Ensure that** **WhatsApp or SMS messaging** **is set up, and participants know that a 6-week acclimatization and support program will be sent to them**			
	Congratulations on your hearing aid	Day 0 (day of fitting)	
**Getting used to a hearing aid, understanding how it works and how to care for it**			
	Inserting and wearing your hearing aids properly	Day 1	
	When and how often to wear hearing aids	Day 2	
	Storing the hearing aid	Day 3	
	Inserting new batteries	Day 4	
	How does the hearing aid work?	Day 5	
	Cleaning and maintenance of the hearing aid	Day 6	
**Troubleshooting**			
	Managing earwax	Day 7	
	Volume and program control	Day 10	
	Self-help strategies if the hearing aid does not produce sound (1)	Day 13	
	Self-help strategies if the hearing aid does not produce sound (2)	Day 15	
**Optimal hearing aid use**			
	Hearing aid do’s	Day 18	
	Hearing aid don’ts	Day 23	
	Using the hearing aid with a phone	Day 25	
	Using the hearing aid with a mask	Day 28	
	Environmental adaptations	Day 30	
	How to make hearing aid batteries last longer	Day 33	
**Confidence using hearing aids (recap most important factors)**			
	How to use the drying capsule	Day 35	
	Adjusting to the hearing aid	Day 38	
	Congratulations on completing the program	Day 40	

^a^mHealth: mobile health.

^b^English summary of isiXhosa messages.

##### Translation

The 20 unique English messages were translated and adapted to isiXhosa, the home language in the target communities. Two CHWs, employed by the NGO partner, whose first language is isiXhosa, and are fluent in English, were recruited to assist with the translations. The CHWs worked independently to translate the messages and then produced a consolidated isiXhosa translation. The original English and isiXhosa versions of the first 7 messages were sent to a certified isiXhosa linguist. The linguist compared the translations and suggested edits. The CHWs then reviewed these edits to ensure clarity according to the prevailing dialect used in the communities. On the basis of the high accuracy of their translations, CHWs translated the remaining messages without assistance from the linguist.

##### Presentation Format

**Figure 1:**
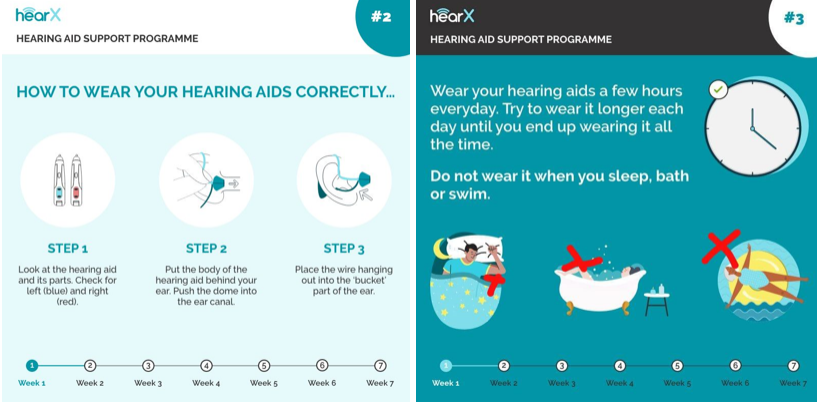
Examples of graphical illustrations with textual explanations were accompanied by a voice note and sent to participants who had access to WhatsApp.

The content of the 20 messages was used to create 20 voice notes sent via WhatsApp or 20 SMS text messages if WhatsApp was unavailable. Voice notes were used to ensure comprehension regardless of the users’ literacy level, and voice notes also have an option of increasing the volume if needed. The voice notes ranged from 10 to 93 seconds and were accompanied by graphical illustrations with textual explanations in English to summarize the information provided (Figure 1).

The 20 unique messages were sent over a period of 45 days, with all participants receiving the same messages on the same days. Week 1, the primary focus for acclimatization, included 8 messages. Messages in subsequent weeks were spread to include two in weeks 2 and 3, three in weeks 4 and 5, and two in week 6 (Table 1).

#### Phase 2—Implementation and Pilot Testing of the mHealth Acclimatization and Support Program

##### Recruitment

Community members of 2 low-income communities received a CBR service providing hearing aids to adults previously reported by Frisby et al [25]. These communities were identified by the NGO partner, which previously worked with community leaders and identified a need for hearing services in these communities. The average monthly household income in the 2 communities, Khayelitsha and Mbekweni in the Western Cape, South Africa, is less than ZAR 3183.3 (US $202) for 74% and 76% of households, respectively [53,54]. Three CHWs, 33, 38, and 46 years of age, fluent in both isiXhosa and English, and members of these communities, were recruited to facilitate this service. The CHWs previously received training through the NGO partner on hearing screening services for children [20] and adults [25] in the community. CHWs received an additional 2 days of training by an audiologist facilitated by the NGO partner before the commencement of this study. Training covered included the features and functionalities of the hearing aids, measurement of tube length, hearing aid insertion, maintenance of hearing aids, and training on how to instruct participants on the use and maintenance of their hearing aids.

##### Procedures

Eligible participants met the following inclusion criteria: (1) age of ≥18 years, (2) bilateral hearing loss (pure tone average ≥26 dB hearing loss [55] with thresholds ≤85 dB hearing loss at any frequency [maximum output of hearing aids]), (3) normal middle ear functioning determined through visual inspection via a digital otoscope, (4) no previous experience with hearing aids, (5) able to receive program messages (themselves or a household member), and (6) willing and able to purchase batteries monthly beyond the life of the project (approximately ZAR 50 [US $3.4] per pack at pharmacies). No exclusions were made based on cognitive indices or literacy levels.

The hearing aid fittings took place during a second home visit while the audiologists observed (observation was purely for data collection purposes). The audiologists were not involved in providing the program or collecting the data. These digital hearing aids were fit via Bluetooth through a smartphone according to a standard hearing aid fitting algorithm (ie, NAL-NL2) using participant hearing thresholds. The hearing aids were behind-the-ear (Lexie Lumen), slim tube, non-custom mold devices with 16 channels, wide-dynamic-range compression technology, feedback reduction, Bluetooth connectivity and programming, digital noise reduction, and a directional microphone array [25,48]. Once fitted, the hearing aids did not require a phone for continuing operation. CHWs orientated the participants on user-operated controls and device maintenance. Participants were given accessories to last the study duration (eg, a drying kit, tubes, domes, and batteries).

**Figure 2:**
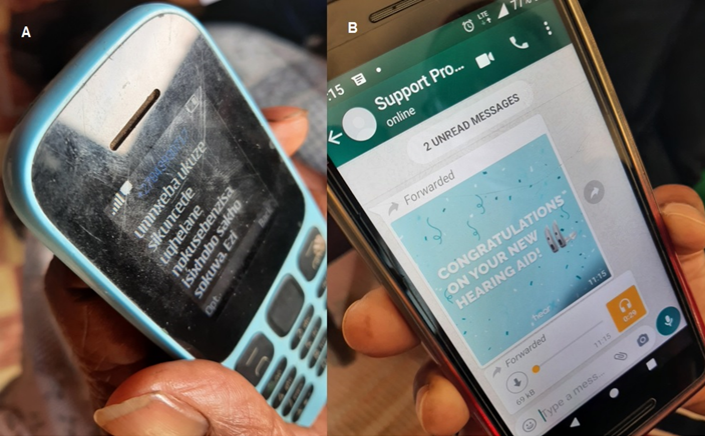
(A) Participant receiving the first acclimatization and support program message via SMS text messaging. (B) Participant receiving the first acclimatization and support program image and voice note via WhatsApp.

Participants were enrolled in the mHealth acclimatization and support program. CHWs were supplied with project smartphones (Android) and were responsible for sending the messages to participants. Participants who did not have access to WhatsApp received the program through SMS text messages. Participants were provided with the CHWs’ contact details and could contact the CHWs if they experienced any difficulties. Participants accessing WhatsApp received graphical illustrations with textual explanations accompanied by a voice note (Figure 2).

##### Outcome Measures

Participants completed a nonstandardized, closed-ended questionnaire before initiating the acclimatization and support program to determine their competence in operating a mobile phone (Multimedia Appendix 1). Subsequently, CHWs telephoned participants on days 8, 20, and 43 of the program. Open-ended questions were asked in isiXhosa to enquire about possible challenges or issues with the hearing aids (Multimedia Appendix 1). CHWs made written notes of participant responses during the phone calls.

**Figure 3:**
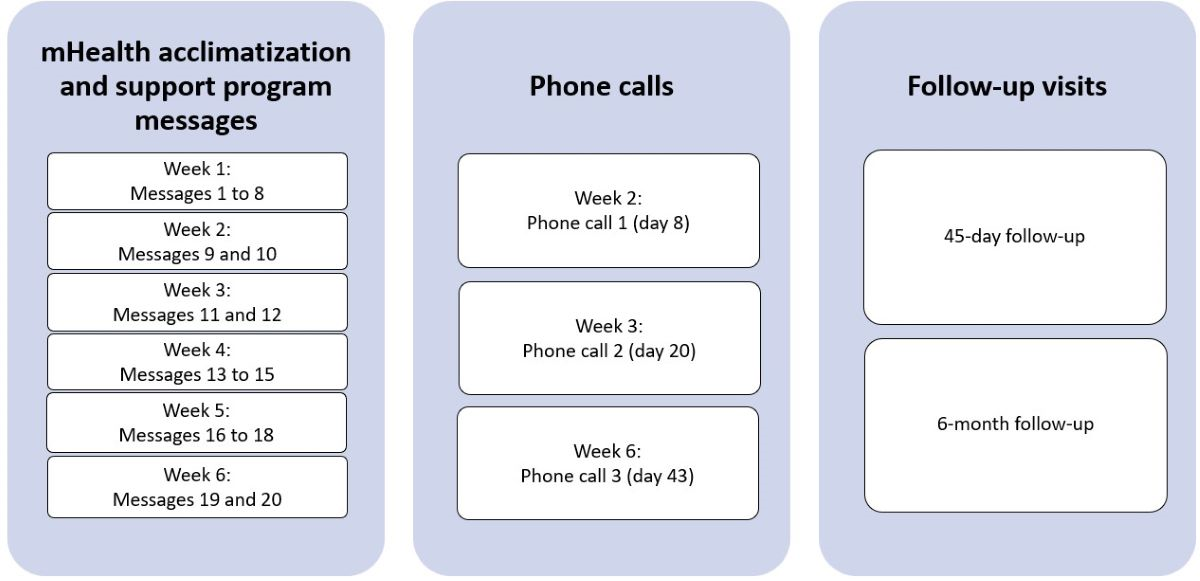
Timetable of the various types of support provided to the participants by the community health care workers during the acclimatization and support program. mHealth: mobile health.

CHWs conducted home visits 45 days and 6 months after the acclimatization and support program started. Participants completed a nonstandardized, open-ended questionnaire (Multimedia Appendix 1) at both follow-up visits to obtain qualitative information on their experiences, perceptions, and accessibility of the acclimatization and support program. Participants were also asked which of the messages they reviewed several times. All questionnaires were translated to isiXhosa. All questionnaires were answered in an interview format with the CHWs. Various types of support provided to participants during the acclimatization and support program are shown in a timetable in Figure 3.

### Data Analysis

Raw data were recorded in an Excel 2016 (Microsoft Corp) spreadsheet and imported for statistical analysis into the SPSS software (version 27; IBM Corp). Observational descriptive analysis, including mean, frequency counts, and SDs, were determined for quantitative statistics such as participant age, gender, and degree of hearing loss. Qualitative open-ended questionnaire responses were analyzed using inductive thematic analysis with a semantic approach [56]. To ensure trustworthiness and credibility, the first author (CF) applied a rigorous and systematic process to data analysis. The first author (CF) familiarized herself with the data through transcription and by recording the raw data onto an Excel spreadsheet, generated initial codes, and performed a qualitative analysis to identify and categorize broad themes. To enhance credibility and reduce bias, this analysis was independently reviewed and vetted by the last author (DWS), who served as a critical reader. Through this collaborative review process, any potential biases or misinterpretations were addressed, ensuring that the data were accurately represented and that the analysis was robust and reliable.

## Results

### Phase 1—Development of the mHealth Acclimatization and Support Program

In total, 20 messages were adapted and developed from the Lexie Hearing application (Lexie Hearing; Table 1). The mean readability score of all English language messages was 85.3 (SD 9.4, range 55-96.5), with a mean grade-level score of 5.4 (SD 0.9, range 4-7).

### Phase 2—Implementation and Evaluation of the mHealth Acclimatization and Support Program

A total of 19 participants fitted with hearing aids received an mHealth acclimatization and support program over 45 days. Participants’ ages ranged from 48 to 96 (mean 71.7, SD 13) years. Females accounted for 78.9% of participants. Participant hearing status and competence in operating a mobile phone are reported in Table 2. A third of the participants (6/19, 32%) needed assistance to operate a phone. Most participants (15/19, 79%) received the program via WhatsApp, and 21% (4/19) elected to receive this program via SMS text message.

**Table 2:** Hearing status of participants fitted with hearing aids and their reported ability to use a phone (N=19).

Hearing status		Value
**Hearing (0.5, 1, 2, and 4 kHz PTA^a^)**		
	Mean 4-frequency PTA, dB HL^b^ (SD), left	44 (13)
	Mean 4-frequency PTA, dB HL (SD), right	46 (13)
	PTA, dB HL range, left	26-74
	PTA, dB HL range, right	25-68
**Degree of HL (better ear PTA), (%)**		
	Mild (25-40 dB HL)	8 (42)
	Moderate (41-60 dB HL)	9 (47)
	Moderately severe (61-80 dB HL)	2 (11)
**Duration of self-reported hearing loss (years), n (%)**		
	1-5	11 (58)
	6-10	1 (5)
	11-20	5 (26)
	>20	2 (11)
**Please select the most appropriate option regarding how comfortable you are operating a phone (close-ended), n (%)**		
	I know how to make and receive calls	3 (16)
	I can make and receive calls and send and receive text messages via SMS	3 (16)
	I can make and receive calls and send and receive text messages via WhatsApp	7 (37)
	I need someone at home to help me use the phone	6 (32)

^a^PTA: pure tone average.

^b^HL: hearing loss.

Overall, 14, 7, and 15 participants could be contacted during phone calls 1, 2, and 3, respectively, and all reported using the program (ie, receiving the messages and then reading the messages, listening to the voice notes, or viewing the images). Most participants who could be contacted for phone calls 1, 2, and 3 (n=12, n=6, and n=14, respectively) were satisfied with the hearing aids and program. Four participants had a complaint regarding the hearing aids during the 3 phone calls. This included feedback (n=1), pain (n=1), hearing aids falling out (n=1), hearing aids not working, and confusion regarding the drying kit (n=1). How these hearing aid issues were addressed is reported in Table 3. No participants contacted the CHWs with questions or concerns outside of the 3 phone calls.

**Table 3:** Manner in which CHWs^a^ addressed the hearing aid issues reported during follow-up phone calls (days 8, 20, and 43 of the acclimatization and support program).

Category of issues	Number of complaints (n)	How CHWs addressed the issue
Hearing aid performance	2	Feedback: During a home visit, CHWs determined that feedback was due to a headscarf blocking hearing aid and incorrect dome size (too large). The dome was replaced, and CHWs successfully counseled the participant. Battery performance: CHWs determined that the batteries were rapidly draining and thus flat due to being stored in the drying kit. CHWs successfully counseled the participant on correct battery storage.
Physical fit	2	Pain: CHWs determined that the participant put the incorrect tube on the hearing aid (left tube placed on the right hearing aid). CHWs successfully replaced the tube and counseled the participant. Hearing aid falling out of ear: CHWs counseled the participant on correct placement of the hearing aids by using a mirror when inserting the hearing aids.
Hearing aid accessories	1	Drying kit confusion: CHWs successfully instructed the participant on drying kit usage.

^a^CHW: community health care worker.

Overall, 17 participants took part in the 45-day follow-up and 16 in the 6-month follow-up. All participants reported making use of the program and felt a general sense of support provided by the program. Most participants (11/17, 65%) relied on others in their homes to receive the information as they did not own phones or could not operate a phone independently. Of these participants, 64% (7/11) relied on their children, 18% (2/11) on their spouses, and 18% (2/11) on grandchildren. Participant responses to open-ended questions asked at the follow-up visits are reported in Table 4.

**Table 4:** Thematic analysis of responses to open-ended questions by participants with hearing aids at the 45-day follow-up visit (n=17) and the 6-month follow-up visit (n=16).

Themes		Frequency	Example responses
**How do you feel about the services you received in the program? Did the WhatsApp or text message give you enough information? (45-day follow-up)**			
	Helpful, supportive, and informative	6	“The messages were very helpful, especially the Do’s and Don’ts.” “The service was helpful. The messages were clear and reminded them of everything said.” “Felt well supported.” “Very happy that I received the messages. I learned a lot of things that I did not know from the messages. Good info. The program is fruitful.”
	Sufficient and clear information	11	“I loved the service received because it gave us information that we did not have. I think that the information on the text message was enough.” “I am grateful for the service that I received, and the information received on WhatsApp and text message was enough for me.” “Info was enough and clear. I liked the program, especially the voice notes.” “It served as a reminder of the fitting. Info was enough. It was very good.”
**In what way was the information helpful? (6-month follow-up)^a^**			
	Support and information given	10	“Yes, it explained everything. It was helpful and clear.” “Yes, whenever I needed help, I could listen to the voice notes, and they would help.” “Yes, it was very helpful, and the information was clear.”
	Cleaning hearing aids	3	“Yes, it helped a lot to teach me how to clean the hearing aids.” “It was very helpful, taught me how to clean my hearing aids.”
	Changing batteries	2	“Yes, it helped me learn how to change batteries.” “Yes, very helpful, especially on how to change the batteries.”
	Could not recall	2	“I cannot recall the information or if it was helpful.” “I do not remember the information from WhatsApp.”

^a^n=17 as 1 participant reported that the information was helpful across 2 themes.

All participants (n=17) reported feeling well supported and that messages were easy to understand. Most participants (14/17, 82%) reported that the program messages covered all questions about their hearing aid. A total of 7 participants reported difficulties performing specific tasks with the hearing aids, including difficulties putting on the hearing aid on (n=3), cleaning the hearing aid (n=3), and changing the batteries (n=1). These reported difficulties were covered by the program messages, and these participants reported that the information provided in the program made these tasks easier. Only 3 of 17 (18%) participants reported that the acclimatization and support program did not answer all their questions (drying kit color change, where additional capsules could be bought, how and when to change the dome of the hearing aid). Almost half (8/17, 47%) of participants reported that they reviewed the program messages several times. Two participants reported that although they did not experience any difficulties, they reviewed all messages as a general reminder of the concepts covered.

Among the 16 participants who participated in the 6-month follow-up visit (Table 4), 14 reported that they were still using their hearing aids. Three participants reported reviewing the messages since the 45-day follow-up. Some general comments the participants made about the acclimatization and support program include “I liked that I could refer back to the messages - for example, how to clean the hearing aid,” “The text messages gave me advice on what to do. I loved them because they are in isiXhosa,” “I am forgetful as a result of my age and often forget things and will ask for the messages to be shown to me again,” and “Initially I struggled but used pictures to help, and now I’m fine.” Participants were asked an open-ended question on their perceptions regarding any improvements that could be made to the program. Although the overall responses were positive, 2 of 16 (13%) participants indicated that additional information might be required to improve the program’s content (expected lifespan of the hearing aids, if a family member could receive the hearing aid should the participant pass away, permanency of their hearing loss, and how long the use of the hearing aids would be necessary). An additional 3 participants reported that improvements could be made to the program. These included increasing the volume of voice notes and increasing the text size on graphical illustrations (n=1), and additional home visits by the CHWs to provide hands-on training on hearing aid use and maintenance to supplement the messages (n=2).

## Discussion

### Principal Findings

This study implemented and evaluated an mHealth acclimatization and support program for first-time hearing aid users in low-income communities, facilitated by CHWs. The mHealth program aimed to improve first-time hearing aid users’ usage of their hearing aids by providing basic information covering how a hearing aid works, optimal use and care, troubleshooting, and motivational support. The feasibility of the mHealth acclimatization and support program in this study could be ascribed to several factors, including the accessible information with text developed with lower reading grade levels and accessibility of the platforms used to distribute the program. The readability level of information is critical for ensuring accessible health literacy [57,58]. To ensure patient understanding, it is recommended that the content of health care education materials should not exceed a grade level of more than US grade 5 [57,59]. A recent review examining the readability of patient education materials, including brochures and user guides, found that most of the average reading grade level across the studies ranged from 5.05 to 11.4, which exceeds the recommended reading grade levels [58]. The average reading grade level of the English-language messages used in this program was US grade 5.4. The same level was maintained during translation, which may have contributed to participants’ ease of understanding. Furthermore, most of the participants were bilingual in isiXhosa and English. If not, a significant household member was bilingual to ensure that all participants understood the English textual explanations in the graphical illustrations.

Using an mHealth platform is relevant because nearly 5.7 billion individuals globally are predicted to have access to mobile phones by 2025, of which approximately 84% will be smartphones [60]. Although the penetration rate of mobile phones is relatively high and will continue to increase, most (11/17, 64.7%) of the older participants reported that they were either unable to operate a mobile phone independently or did not own a mobile phone. This challenge was addressed through household members receiving the mHealth program and conveying the information to participants. All participants received the same messages on the respective days of the program, which ensured consistency across study participants. Moreover, as the messages were provided via mobile phones, participants were able to revisit messages. Several participants reviewed the program messages multiple times to revisit concepts they may have felt uncertain about or needed reminding of. The option to revisit messages is a benefit as first-time hearing aid users often report difficulty with the vast amount of information provided during a fitting appointment [27]. This program will likely enhance information retention. Additionally, each message targeted only one specific concept of hearing aid use and management. Therefore, if participants had difficulty with one message, they would not fall behind on other concepts.

The positive reports by participants in this study, indicating an experience of being supported by the program, compare well with other studies [25,37,38]. For instance, 88% of hearing aid users who received an education program via DVD or the internet reported positive experiences [61], and 84% of first-time hearing aid users who received an mHealth educational program accessed via the internet reported feeling more confident with hearing aid use and communication [42]. This study is, however, distinct as it is the first mHealth acclimatization and support program offered via messaging platforms and using the community-based hearing rehabilitation framework [62]. A recent review found that mHealth facilitated by CHWs supported and improved patient outcomes when used for education and supplying information [63]. The results of this feasibility study suggest a similar effect on hearing aid acclimatization and support, but further investigations need to determine the contribution of the mHealth support program to hearing aid use and outcomes using controlled studies.

This study demonstrates that mHealth technologies facilitated by CHWs in low-income communities can support the rehabilitation process of hearing aid users and improve hearing aid use. This study also determined that audiologists are not required in the field during the implementation of such a program and that audiologist-led training and remote support via teleaudiology is sufficient. Although the overall responses were positive, it may be beneficial for future studies to consider incorporating additional information on hearing loss and its permanency, the lifespan of hearing aids, how each hearing aid is personalized for the user, usage of a drying kit, and how to replace domes or tubing.

### Limitations

CHWs were assigned several participants and had to track when to send the messages to each participant. Although no errors were reported in this study, this is a barrier to scaling the intervention. Future studies and implementations should employ automated programs to allow for improved scalability and a reduced chance of human error. The graphical illustrations sent via WhatsApp contained textual explanations that were in English. Future research should consider providing all information in participants’ home language. This study did not perform user testing on the graphical illustrations before implementation, which will be useful in future to ensure the appropriateness and clarity of the images, messages, and font style and size. Future research should consider using a pre-post measurement of practical skills needed to use and maintain hearing aids to investigate postintervention changes. Future studies should investigate how cognitive functioning and literacy levels could influence the benefit of such an intervention. This study had a small sample size, and female participants were overrepresented. Furthermore, objective measures were not included to determine if participants accessed the program or hearing aid use. This study made use of observational descriptive analysis for the quantitative data; future research on larger sample sizes will allow inferential statistics. Most importantly, owing to the feasibility nature of this study, the results should be seen as preliminary. Controlled studies are needed to evaluate the outcome and the effect size of such programs. A cost-effectiveness study is needed to determine the usability of such a program in a real-world context.

### Conclusions

An mHealth acclimatization and support program for first-time hearing aid users supported by CHWs was adapted for use in LMICs and implemented successfully. Feedback from the hearing aid users was positive, with all participants feeling well supported, and most were still making use of their hearing aids 6 months later. Making use of a real-world sample of participants illustrated that an mHealth acclimatization and support program provided in the home language of the users is feasible and can assist self-management of first-time hearing aid users in LMICs. These results demonstrate the potential for self-management of hearing aids from first-time hearing aid users’ homes. This is particularly relevant in LMICs where, owing to a lack of resources and professionals, opportunities for in-person interactions with hearing health care professionals may be limited. Scalable mHealth support options can facilitate increased access and quality in hearing care. Future research should consider randomized controlled trials to compare hearing aid self-management outcomes with and without the support program.

## Data Availability

The data sets generated during and/or analyzed during this study are available from the corresponding author on reasonable request.

## Appendix

Multimedia Appendix 1 Questionnaire.

## Abbreviations

CBR: community-based rehabilitation
CHW: community health care worker
LMIC: low- and middle-income country
mHealth: mobile health
NGO: nongovernmental organization
