# Supplementary material for: Mobile Health Hearing Aid Acclimatization and Support Program in Low-Income Communities: Feasibility Study
Source: JMIR Form Res. 2023 Aug 23;7:e46043. doi: 10.2196/46043 (PMC10483300; doi:10.2196/46043)
Supplement: Multimedia Appendix 1 [file formative_v7i1e46043_app1.docx]

Multimedia Appendix 1

**Prefitting Questionnaire**

1. Which of the following cases (sentences/statements) best describe how you feel about your hearing at the moment?

1. I do not think I have a hearing problem, and therefore, nothing should be done about it.
2. I think I have a hearing problem. However, I am not yet ready to take any action to solve the problem, but I might do so in the future.
3. I know I have a hearing problem, and I intend to take action to solve it soon.
4. I know I have a hearing problem, and I am here to take action to solve it now.

2. How do you feel about wearing a hearing aid?

1. I feel excited.
2. I feel ok to wear a hearing aid.
3. I feel scared.
4. I feel ashamed or embarrassed.

3. Do you know of someone who wears a hearing aid?

1. Yes If yes, who?......................................................
2. No

4. For how many years have you had a problem with your hearing?

__________________________________________________________________________________________________________________________________________________

5. How has your hearing problem affected your life?

__________________________________________________________________________________________________________________________________________________

6. How do other people treat you because of your hearing problem?

__________________________________________________________________________________________________________________________________________________

7. How comfortable are you in using a phone?

1. I can receive phone calls.
2. I know how to make and receive calls.
3. I can make and receive calls and send and receive text messages via WhatsApp.
4. I need someone at home to help me in using the phone.

**Script for phone calls**

**Phone Call One: 8 days after hearing aid fitting**

Hello. My name is........ I am calling from the hearing aid support programme. Well done on wearing your hearing aids for a week! I am calling to find out how it is going? Is this a good time to talk?

• Did you have any difficulty wearing the hearing aids? If yes, what did you struggle with?

• Are you managing to put the hearing aids into your ears? (Or with support from family members?)

• Do you understand how to clean the hearing aids?

• Did you understand the WhatsApp/SMS messages?

• How do the hearing aids sound? (Is it too loud? Are you happy with it?)

• The hearing aid may sound uncomfortable at first because your brain is hearing sounds that it has not heard in a long time. The first part of getting used to it is the hardest but as you wear it more often, the sound will be more comfortable.

**Phone Call Two: 20 days after hearing aid fitting**

Well done on wearing your hearing aids for three weeks! You have now learnt about common problems that happen with hearing aids, and how it can be fixed.

• Have you experienced any problems with your hearing aids?

• Are you getting used to how the hearing aids sound?

• Can you clean the hearing aid?

• Have you heard the "battery low - replace battery" message? Did you put in new batteries? [By now they should have changed the batteries]

**Phone Call Three: 43 days after hearing aid fitting**

Hello! I am (name), calling from the hearing aid support programme. It has been more than 40 days since you got your hearing aids. I am calling to find out how it is going and to hear if you were happy with all the information.

• Do you feel comfortable wearing your hearing aid?

• Are you managing to clean the hearing aid?

• Are you having any problems with wax or dirt in the dome or tube? (The hearing aid will sound blocked or funny.)

• Are you finding anything difficult with your hearing aid? (for example, difficult to change batteries, difficult to hear over the phone?)

• Is there anything you need help with for your hearing aid?

• How often are you wearing your hearing aid?

• I would like to know how this programme was for you. Do you feel like the messages, calls, and pictures have helped you get used to your hearing aid? If not, why? What could we have done better?

• Can we come for a final home visit to check how it is going with the hearing aid and ask some more questions?

**45-day follow-up Questionnaire:**

1. Which of the cases best describe how you feel about your hearing at the moment?

I do not think I have a hearing problem, and therefore, nothing should be done about it.

I think I have a hearing problem. However, I am not yet ready to take any action to solve the problem, but I might do so in the future.

I know I have a hearing problem, and I intend to take action to solve it soon.

I know I have a hearing problem, and I am here to take action to solve it now.

2. What was your experience of the hearing aid support programme?

I felt well supported.

The support provided could have been better.

The support was of no use.

Any Comments?

3. Did you find it difficult to perform any of the following tasks?

Putting the hearing aid on

Cleaning the hearing aid

Putting in new batteries

Storing the hearing aid

4. How do you feel about the service you received in the programme? Did the WhatsApp/SMS give you enough information?

__________________________________________________________________________________________________________________________________________________

5. Were you able to receive the messages every time they were sent or did you only get some later due to not having data?

__________________________________________________________________________________________________________________________________________________

6. Did you ever go back and reread the messages or relisten to the voice notes to remind you how to use your hearing aid? If yes, which one?

__________________________________________________________________________________________________________________________________________________

7. Was the information in the WhatsApp/SMS easy to understand?

__________________________________________________________________________________________________________________________________________________

8. Were there times that you did not wear your hearing aid? Why?

__________________________________________________________________________________________________________________________________________________

9. Would you have preferred if the pictures were in your home language (Xhosa)?

__________________________________________________________________________________________________________________________________________________

10. Did you ever have any problems with the hearing aids but did not have data or airtime to contact the community healthcare workers?

__________________________________________________________________________________________________________________________________________________

11. Only for the participants with WhatsApp: Did you find the picture, or the voice note easier to understand? Which did you prefer?

__________________________________________________________________________________________________________________________________________________

12. Do you have any questions about how to use or look after your hearing aid that the messages didn't answer?

__________________________________________________________________________________________________________________________________________________

13. For the SMS participants: Was the SMS easy to read or would you have preferred to get a picture or a voice message?

__________________________________________________________________________________________________________________________________________________

14. Any recommendations to improve the support programme

__________________________________________________________________________________________________________________________________________________

**6-month follow-up Questionnaire**

1. Are you still using your hearing aids? YES/NO

If YES above - Can you share how the hearing aids have impacted your life? (e.g., Hear the pastor better in church, can hear better over the phone during phone calls, can hear cars better when going for a walk, etc.)

__________________________________________________________________________________________________________________________________________________

2. If YES - How often do you wear your hearing aids? (i.e., every day, couple of days per week, once a week, only for certain events)

__________________________________________________________________________________________________________________________________________________

3. IF NO above - Can you share why you are not using your hearing aids anymore?

__________________________________________________________________________________________________________________________________________________

4. Are you experiencing any difficulties with your hearing aids and if so please explain?

__________________________________________________________________________________________________________________________________________________

5. Are you managing to change the batteries? How often do you change your batteries?

__________________________________________________________________________________________________________________________________________________

6. Have you changed the tubes/domes?

__________________________________________________________________________________________________________________________________________________

7. Are you managing with cleaning the hearing aids? How often do you clean your hearing aids? Who cleans it?

__________________________________________________________________________________________________________________________________________________

8. Do you have any concerns about wearing your hearing aids?

__________________________________________________________________________________________________________________________________________________

9. Would you recommend hearing aids to other people with hearing difficulties? Why/Why not?

__________________________________________________________________________________________________________________________________________________

10. If you think back to the information you received via WhatsApp/SMS after getting the hearing aids, was the information helpful and if so in which ways? __________________________________________________________________________________________________________________________________________________

11. Did you ever go back to the WhatsApp/SMS messages since our last visit? If so which ones?

__________________________________________________________________________________________________________________________________________________

12. After having your hearing aids for 6 months do you have any new questions about how to use or take care of your hearing aids?

__________________________________________________________________________________________________________________________________________________
